# Fatty Acid Variation in Macadamia Kernels: Association of Palmitoleic Acid with Fruit Traits and Selection of Multi-Attribute Germplasm

**DOI:** 10.3390/foods15213877

**Published:** 2026-10-29

**Authors:** Yang Li, Fan Yang, Nanhua Zi, Tingyu Li, Jing Ma, Lidan Gong, Ziqiang Li, Chao Wu, Jianjian Geng, Qingyi Long, Liang Tao

**Affiliations:** 1 Yunnan Institute of Tropical Crops, Jinghong 666100, China; yangshi0503@126.com (Y.L.); zinanhua123@163.com (N.Z.); tingyuli111@outlook.com (T.L.); majing160101@163.com (J.M.); gld2001@126.com (L.G.); dcdsylzq@126.com (Z.L.); m13669757704@163.com (C.W.); genggengjian@163.com (J.G.); lqingyi@163.com (Q.L.); 2 Institute of Tropical and Subtropical Cash Crops, Yunnan Academy of Agricultural Sciences, Baoshan 678000, China; yflyxy@126.com

**Keywords:** macadamia, palmitoleic acid (C16:1), fatty acid composition, germplasm, entropy-weighted TOPSIS, fruit traits, breeding

## Abstract

Palmitoleic acid (C16:1) is rare among woody oil crops yet accumulates to high levels in macadamia kernels, where its inter-accession variation and relationship with fruit traits remain poorly characterized. We profiled 22 fatty acids by GC-MS, quantified crude fat, crude protein and total soluble sugar by NIRS, and measured nine fruit traits in 139 accessions. The major fatty acids ranked as C18:1 > C16:1 > C16:0. C16:1 varied widely among accessions (mean 10.66%, CV = 27.7%, range 2.78–19.05%), about 12-fold the analytical RSD, whereas C18:1 was stable (CV = 5.3%). C16:1 was strongly negatively correlated with C18:1 (r = −0.921, *p* < 0.001) and positively correlated with C16:0 (r = 0.534). Correlation, partial correlation and PCA consistently indicated that fatty acid composition was independent of fruit traits; after controlling for C18:1, C16:1 retained only a weak positive association with single dry kernel weight (r = 0.170, *p* < 0.05). Entropy-weighted TOPSIS with five indicators selected five accessions combining high C16:1 with desirable fruit traits. These accessions provide breeding material for C16:1-enriched oil, and the independence of C16:1 from fruit traits means that both can be improved simultaneously.

## 1. Introduction

Macadamia (*Macadamia* spp.) is highly valued for its unique flavor and high nutritional properties [1]. The kernel has an extremely high oil content, accounting for approximately three-quarters of its mass, making it an important source of high-quality vegetable oil [2]. At present, China accounts for more than one-third of the global macadamia planting area, and the industry is developing rapidly [3,4]. Studies have shown that macadamia oil is rich in unsaturated fatty acids (approximately 78%, *w*/*w* of dry kernel) and contains bioactive components such as phytosterols, squalene, and polyphenols, with high nutritional value and health benefits [5,6].

Fatty acid composition is an important indicator for evaluating the oil quality of nuts. Approximately 80% of the fatty acids in macadamia kernels are monounsaturated fatty acids (MUFA), mainly oleic acid (C18:1, ~60%) and palmitoleic acid (C16:1, ~20%), and the major fatty acids ranked as C18:1 > C16:1 > C16:0 [2,7,8]. The C16:1 content reported in different studies ranges from 7.0% to 36.0%, exceeding that of most vegetable oils [5]; four macadamia cultivars grown in New Zealand (Jordan, PA 39, Beaumont, and GT 207) showed an average MUFA content of approximately 80%, with oleic acid at 40.6–59.0% and palmitoleic acid at 16.9–33.8% [9]. Macadamia has one of the highest MUFA contents among natural foods, exceeding 60 g per 100 g of whole edible nut [10].

C16:1 is an omega-7 monounsaturated fatty acid that is rare in the plant kingdom. Compared with other edible nuts and oilseeds (usually less than 6%), the C16:1 content in macadamia reaches 15–22%, making macadamia one of the few plants rich in C16:1, together with sea buckthorn (*Hippophae rhamnoides*) and durian (*Durio zibethinus*) [11]. Among common woody oil crops, the MUFA of almond and hazelnut (*Corylus heterophylla*) [12], *Camellia oleifera* [13], and avocado (*Persea americana*) [14] consists almost entirely of oleic acid (69.24%, 79.30%, 76.73%, and 61.18%, respectively), with low C16:1 contents (0.63%, 0.29%, 0.12%, and 9.82%, respectively), and only macadamia contains approximately 17% C16:1 [12]; coconut (*Cocos nucifera*) oil contains only 0.05–3.90% C16:1 [15]. Therefore, the high C16:1 content is an important characteristic that distinguishes macadamia oil from other nut oils [8].

C18:1 and C16:1 are beneficial to cardiovascular and cerebrovascular health. Studies have shown that high MUFA intake is associated with a reduced risk of coronary heart disease [5], and C18:1 and C16:1 are essential for lowering plasma low-density lipoprotein (LDL) cholesterol and preventing cancer and atherosclerosis [8]. Supplementation with or addition of macadamia to cholesterol-lowering diets significantly reduced LDL-C concentrations by 4.0–10.7% [16], and a macadamia-rich diet was almost as effective as a moderate low-fat diet in lowering plasma total cholesterol and LDL cholesterol [10]. Animal experiments showed that macadamia oil activated the AMPK signaling pathway, reduced serum and hepatic total cholesterol, triglycerides, and LDL-C levels, and increased HDL-C levels [17]. Dietary palmitoleic acid is also associated with a reduced risk of diabetes, inflammation, and cardiovascular disease, and can alleviate oxidative stress and cellular inflammation through the NRF2/TBK1 signaling pathway [11,18].

Fatty acid composition is mainly controlled by genetic factors: in olive (*Olea europaea*) oil, the cultivar factor explained 53.5% of the MUFA variation [19]; fatty acid composition differs markedly among macadamia cultivars, with C16:1 varying from 10.82% to 29.64% among 22 cultivars [20] and from 15.9% to 24.0% among 14 cultivars and 20 breeding lines [2], indicating that the variation in fatty acid profiles is largely genetically determined and that fatty acid composition can be directionally improved by combining traits of different lines. In 188 fruit bearing wild accessions, macadamia fruit traits showed wide variation, with moderate broad-sense heritability (0.43–0.64) [21], indicating that genetic factors make an important contribution to the variation of fruit traits. A previous study systematically analyzed the fatty acid diversity of 197 macadamia accessions and found highly significant differences in C16:1 content among accessions, as well as significant differences among different groups (commercial cultivars, superior selections, and wild germplasm) [22]. Current studies in China have mostly focused on the comparison of major cultivars and production region evaluation, with C16:1 contents ranging from 13.22% to 17.63% among 15 major cultivars [3] and from 11.40% to 18.73% among different production regions [4,23]. However, for the Chinese germplasm population dominated by seedling selections, the inter-accession variation of C16:1 and its relationship with fruit traits have not been systematically evaluated. In this study, 139 macadamia accessions (including commercial cultivars and Chinese seedling materials) were used to determine the fatty acid composition of kernels by gas chromatography–mass spectrometry (GC-MS), to analyze the variation of C16:1 and its correlation with fruit traits, and to select accessions with high C16:1 content and desirable fruit traits by entropy-weighted TOPSIS, thereby providing a reference for functional cultivar breeding in macadamia.

## 2. Materials and Methods

The experiment was conducted in Jinghong City, Xishuangbanna Prefecture, Yunnan Province, China. The 139 accessions used are conserved in the Jinghong Macadamia Germplasm Repository of the Ministry of Agriculture of China, with 3–5 trees per accession. The accessions originated from Hawaii, Australia, and China; the Hawaiian and Australian accessions are mostly commercial cultivars, whereas the Chinese accessions are mostly seedling selections (Table S1). The repository is located in a northern tropical humid monsoon climate zone, with a maximum temperature of 38.4 °C and a minimum temperature of 10.0 °C in 2025, an annual mean temperature of 21.9 °C, no frost throughout the year, annual rainfall of 1196 mm, annual sunshine duration of 2428.1 h, and an altitude of 550 m. Meteorological data were provided by the Jinghong Meteorological Bureau. All accessions were managed under conventional agronomic practices, which were kept consistent.

### 2.1. Investigation of Fruit Traits

Fully mature fruits (dark brown endocarp) were collected from August to October 2025. For each accession, fruits were collected from three trees, with 20 fruits per tree, and the 60 fruits were combined. For accessions with poor fruit set or severe physiological fruit drop, fruits were collected according to the number actually available, with a minimum of 5 fruits per tree. The number of fruits collected per tree was kept as consistent as possible within an accession. After losses from de-husking and shell cracking, the final number of fruits per tree used for analysis was 14.3 ± 3.2. Twenty fruits were randomly selected from the composite sample for the measurement of morphological traits, and all fruits were measured when the total number was fewer than 20. After measurement, the fruits were combined again and randomly divided into three replicate samples for the determination of kernel recovery and NIRS analysis. A single NIRS measurement requires approximately 8 kernels; when a replicate sample contained fewer than 8 kernels after division, it was divided into two replicate samples. Samples of an accession were collected only when the endocarp had turned dark brown, i.e., at physiological maturity.

After collection, fruit fresh weight (FW/g) was measured immediately; fruit equatorial diameter (FD/mm) and fruit longitudinal diameter (FL/mm) were measured with a digital vernier caliper (accuracy 0.01 mm), and peel thickness (PT/mm) was measured by inserting a thickness gauge from the middle of the ventral suture. After phenotyping all fruits, the peel was cracked to obtain the nut-in-shell. The nut-in-shell were placed on a ventilated rack and air-dried with a high-power electric fan for 7 d, and then dried at 38 °C for 48 h → 45 °C for 48 h → 60 °C for 48 h until the moisture content reached 1–2% (two kernels were dried at 105 °C to constant weight, and the moisture content was calculated from the mass before and after drying). After drying, the nuts-in-shell were cracked using a custom-made macadamia kernel extraction machine (Yunnan Aofu Industrial Co., Ltd., Baoshan, China); the cutter diameter was first set to the maximum (usually 30 mm) and then reduced by 2 mm after each cracking, so as to keep the kernels intact as much as possible while cracking all nuts. The shells and kernels of each sample were collected, and kernels adhering to the shells were scraped off with a sample spoon. The shell weight, intact kernel weight, and total kernel weight (intact + broken kernels) of each sample were then weighed, and single dry nut weight (NW, g), single dry kernel weight (KW, g), and kernel recovery (KR, %) were calculated as follows: NW = (shell weight + total kernel weight)/number of nuts; KW = intact kernel weight/number of nuts; KR = total kernel weight/(shell weight + total kernel weight).

Healthy intact fruits were longitudinally cut from the ventral suture with a small saw, and five fruits were randomly selected from each accession. The longitudinal sections were scanned using a Uniscan LA3500 scanner (UNIS, Beijing, China), and the shell wall thickness at sides (SW/mm) and the shell thickness at the hilum (ST/mm) were measured using ImageJ software (1.54P). The scanning resolution was fixed at 600 DPI, and the scale was set according to the formula: pixels per millimeter = DPI value ÷ 25.4; the measured values were exported in mm.

### 2.2. GC-MS Analysis of Fatty Acid Composition

HPLC-grade methanol, dichloromethane and n-hexane were purchased from Merck (Darmstadt, Germany); analytical-grade sodium hydroxide and hydrochloric acid were purchased from ANPEL (Shanghai, China); and double-distilled water was prepared in-house. Standards and isotope-labelled internal standards (≥98 atom % D, ≥99% chemical purity) were purchased from Sigma-Aldrich (St. Louis, MO, USA). Five isotope-labelled internal standards were used: octanoic acid-d15 (C8:0-d15), undecanoic acid-d21 (C11:0-d21), heptadecanoic acid-d33 (C17:0-d33), ^13^C-labelled linoleic acid (^13^C18:2n6c) and docosanoic acid-d43 (C22:0-d43). Stock solutions of the standards (10 mg/mL) were prepared in MTBE, stored at −20 °C, and diluted with MTBE to the working concentrations before analysis.

Concurrently with the sampling described above, a further 12 fruits were collected from the same three trees (four fruits per tree) for GC-MS analysis of the fatty acid composition. The green husk was removed immediately, the nuts were cracked with a manual nutcracker, and approximately one-third of the kernel from each fruit was scooped into 5 mL cryotubes with a sample spoon, and was then immediately frozen in liquid nitrogen and stored at −80 °C.

Before extraction, the sample in each cryotube was soaked in liquid nitrogen for 3 min and ground to a powder in a ball mill (MM400, Retsch GmbH, Haan, Germany) at 30 Hz for 1.5 min. An aliquot of 50 mg of the well-mixed powder was accurately weighed into an Eppendorf tube, and 700 μL of MTBE-methanol (3:1, *v*/*v*) containing the internal standards was added. The suspension was vortexed at 2500 r/min for 10 min and then ultrasonicated for 15 min in a 4 °C water bath. After centrifugation at 4200 rpm and 4 °C for 5 min, 500 μL of the supernatant was transferred to a glass tube containing 200 μL of 0.05 M sodium hydroxide in methanol, and the solution was evaporated to dryness under a stream of nitrogen. The dried residue was redissolved in 400 μL of 0.4 M NaOH–methanol and incubated at 70 °C for 10 min. After cooling to room temperature, 500 μL of dichloromethane and 200 μL of double-distilled water were added, and the mixture was vortexed at 2500 r/min for 5 min and centrifuged at 4200 rpm and 4 °C for 5 min to separate the reaction products. Then, 300 μL of the upper layer was transferred to a new glass tube containing 300 μL of 2 M HCl–methanol, and the solution was incubated at 70 °C for 20 min to obtain the methyl esters [24,25]. After cooling to room temperature, 500 μL of n-hexane and 300 μL of double-distilled water were added, and the mixture was vortexed at 2500 r/min for 5 min and centrifuged at 4200 rpm and 4 °C for 5 min. The upper layer containing the fatty acid methyl esters (FAMEs) was collected into an injection vial for GC-MS analysis.

FAMEs were analyzed on a gas chromatograph (Agilent 8890, Wilmington, DE, USA) coupled to a triple-quadrupole mass spectrometer (Agilent 7000E). Separation was performed on a DB-5MS capillary column (30 m × 0.25 mm × 0.25 μm; Agilent) with high-purity helium (>99.999%) as the carrier gas. The oven temperature was held at 50 °C for 1.5 min, raised to 170 °C at 30 °C/min, to 200 °C at 15 °C/min, to 220 °C at 5 °C/min and held for 2 min, to 228 °C at 8 °C/min, to 240 °C at 20 °C/min and held for 2 min, to 265 °C at 25 °C/min and held for 2 min, and finally to 280 °C at 30 °C/min and held for 2 min. The flow rate was 1 mL/min, the injection volume was 2 μL with a split ratio of 5:1, and the inlet temperature was 250 °C. For mass spectrometry, the electron ionization source was operated at 230 °C and 70 eV, the transfer line at 280 °C and the quadrupole at 150 °C. Data were acquired in SIM mode after a solvent delay of 4 min.

A pooled quality control (QC) sample was prepared and injected three times during the analytical sequence to monitor instrument stability. FAME standards were used for quantification with internal standard calibration curves, and the results were expressed as mg/g fresh weight. The complete GC-MS workflow, including sample preparation and instrumental analysis, was commissioned from MetWare Biotechnology Co., Ltd. (Wuhan, China). For fatty acids with concentrations below the limit of detection (recorded as N/A in the raw data), the values were replaced with half of the minimum detected value before statistical analysis.

### 2.3. Determination of Crude Fat, Crude Protein, and Soluble Sugar Contents by NIRS

The dried kernels obtained in Section 2.1 were used for near-infrared reflectance spectroscopy (NIRS) analysis. The kernels of each accession were mixed and randomly divided into three portions (approximately 15 kernels each), shredded with a cheese grater, and further chopped with a knife. The NIRS samples were measured immediately after preparation; samples that could not be measured immediately were stored at −4 °C until measurement, and the storage time did not exceed 2 d.

The contents of crude fat (FAT/%), protein (PRO/%), and total soluble sugar (SUG/%) were determined by NIRS. The chopped macadamia kernels were passed through a 0.5 mm sieve, and approximately 5 g of powder was placed in a standard sample cup covering the bottom of the cup. Full-spectrum scanning (400–2500 nm, 2 nm interval) was performed with a FOSS NIRS DS2500 spectrometer (FOSS Analytical, Hillerød, Denmark) in reflectance mode. The prediction models for crude fat, protein, and total soluble sugar were established based on standard reference methods (Soxhlet extraction, Kjeldahl nitrogen determination, and anthrone-sulfuric acid method, respectively). The results were expressed as percentages on a dry basis.

### 2.4. Statistical Analysis

Raw data were recorded using Microsoft Excel 2024. Before GC-MS fatty acid analysis, all fatty acids were converted to relative contents (percentages). Python 3.13 was used for data processing and plotting; the pandas library was used to read the Excel data, and NumPy was used to calculate the mean, standard deviation (SD), coefficient of variation (CV), and maximum/minimum ratio, as well as the relative standard deviation (RSD) of QC samples. The SciPy library was used to perform one-way analysis of variance (ANOVA) for traits with replicated measurements. Before ANOVA, the Shapiro–Wilk and Levene tests were used to examine the normality and homogeneity of variance of the raw data. When the assumptions of ANOVA were not met, a permutation ANOVA, which requires no distributional assumptions, was used to compare means. Pearson correlation analysis was used to examine relationships among traits; partial correlation analysis was further conducted to control for the confounding effects among interrelated fatty acids (e.g., C16:1 and C18:1) when evaluating the correlation between fatty acids and fruit morphological traits. Based on the Pearson correlation matrix among traits, hierarchical clustering (R clustering) of traits was performed using (1 − |r|) as the distance measure and average linkage (UPGMA) to explore the grouping structure of traits. Principal component analysis (PCA) was performed using the scikit-learn library on 15 standardized traits (three major fatty acids, nine fruit traits, and three kernel quality traits) to reveal the variation structure of the accessions, and the first four principal components with eigenvalues > 1.0 were retained for interpretation. Based on the results of R clustering and PCA as well as breeding objectives, key traits were selected, objective weights were determined by the entropy weight method based on the information entropy of each indicator, and the accessions were ranked by TOPSIS according to the relative closeness to the positive and negative ideal solutions based on Euclidean distances [26], so as to select elite accessions (following the standard algorithm, implemented with NumPy and pandas). Matplotlib (v3.10.6) and Seaborn (v0.13.2) library were used to generate bar charts, R clustering heatmaps, and biplots. Means are presented as mean ± SD. Statistical significance in figures and tables is indicated by * *p* < 0.05, ** *p* < 0.01, and *** *p* < 0.001.

## 3. Results

### 3.1. Fatty Acid Composition and Fruit Traits of the 139 Accessions

A total of 22 fatty acids were detected in the 139 accessions. Erucic acid (C22:1) was not detected in 99 accessions (71.2%) and its relative content was extremely low (0.04 ± 0.05%); therefore, it was excluded from further analysis. The fatty acid profile of macadamia kernels is shown in Table 1. Among the detected fatty acids, 14 were saturated fatty acids (SFA), accounting for 7.652% of the relative content; 4 were MUFA, accounting for the highest proportion (88.275%); and 3 were polyunsaturated fatty acids (PUFA), accounting for 4.062%. Thus, MUFA was the predominant fatty acid class in macadamia kernels. The major fatty acids (>1%) ranked as C18:1 > C16:1 > C16:0 > trans-C18:1 > trans-C18:2 > C18:2, which is consistent with most previous studies [2,7,8]; the fatty acid composition of macadamia kernels was dominated by C18:1, C16:1, and C16:0, while the contents of other components were low (<5%).

**Figure 1:**
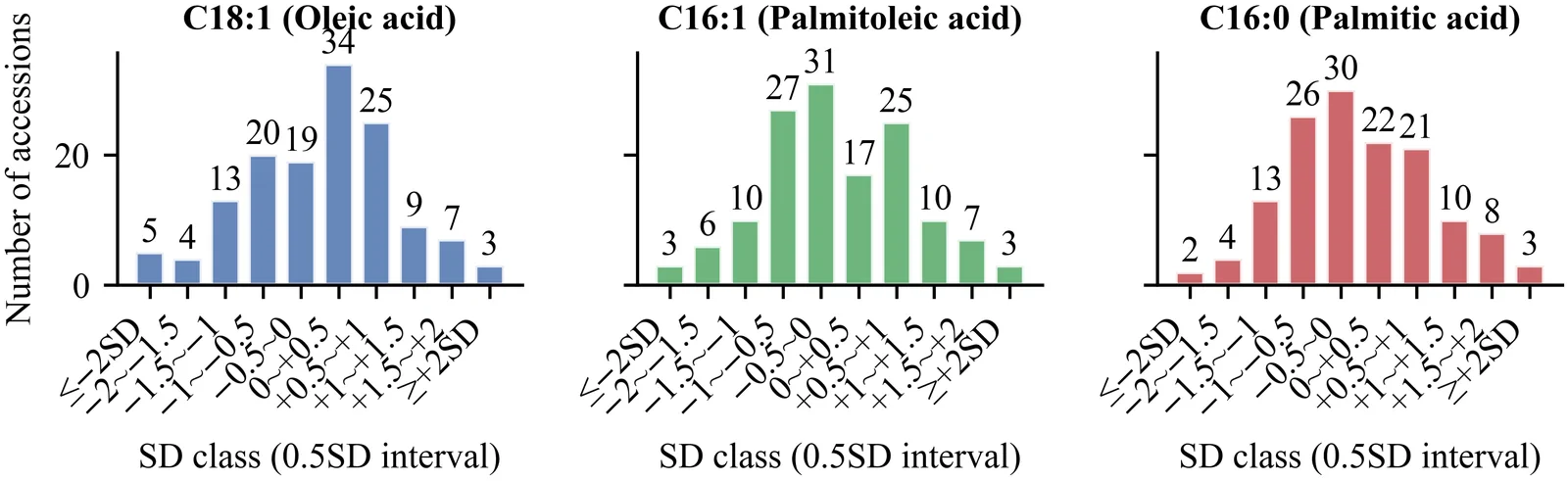
Frequency distribution of the contents of the three major fatty acids in the 139 macadamia accessions classified by mean ± 0.5 SD intervals.

Previous studies have shown that C18:1 and C16:1 are beneficial to cardiovascular and cerebrovascular health, and moderate dietary supplementation can reduce LDL-C levels [5,8,16]. In this study, C18:1 was the most abundant fatty acid with the smallest coefficient of variation, indicating relative stability among accessions; C16:1 is rare in woody oil crops and is present at low levels in *Camellia oleifera*, almond, avocado, and coconut [12,13,14,15]. The C16:1 content of macadamia kernels in this study was relatively high (10.658%), and its CV was 12 times the RSD of the QC sample, indicating certain differences in C16:1 content among accessions. C16:0 was the most important SFA in macadamia, accounting for 75.24% of the total SFA, with a CV of 14.8%, indicating moderate variation among accessions. The Shapiro–Wilk test showed that the three fatty acids were normally distributed (C18:1: W = 0.992, *p* = 0.650; C16:1: W = 0.993, *p* = 0.673; C16:0: W = 0.984, *p* = 0.105). The frequency distributions of the fatty acids classified by mean ± 0.5 SD intervals are shown in Figure 1. C18:1 was concentrated near the mean (±0.5 SD), whereas C16:1 was distributed on both sides of the mean, with the peak spanning −0.1 SD to +1 SD; C16:0 showed a relatively flat distribution with a slight right skew (skewness = 0.35) and an obvious tail toward higher values. The wide distribution and variability of C16:1 contrasted sharply with the concentrated distribution of C18:1, indicating that C16:1 content has potential for genetic improvement.

The assumptions of one-way ANOVA were not fully met: the pooled residuals departed from normality for all 12 traits, and Levene’s test indicated heteroscedasticity for 8 of the 12 traits. A permutation ANOVA, which requires neither assumption, showed that the nine fruit traits and three kernel quality traits of the 139 accessions differed highly significantly among accessions (*p* < 0.001; Table 2). The fruit traits showed moderate variation (5.8–19.2%), among which fruit equatorial and longitudinal diameters varied relatively little, whereas shell thickness at the hilum, fruit fresh weight, and single dry kernel weight varied considerably. Among the kernel quality traits, total soluble sugar content showed the greatest variation, whereas crude fat content showed the least variation (CV = 1.8%); the fat content was relatively stable among accessions but still differed highly significantly.

### 3.2. Interrelationships Among Fatty Acids and Fruit Traits

**Figure 2:**
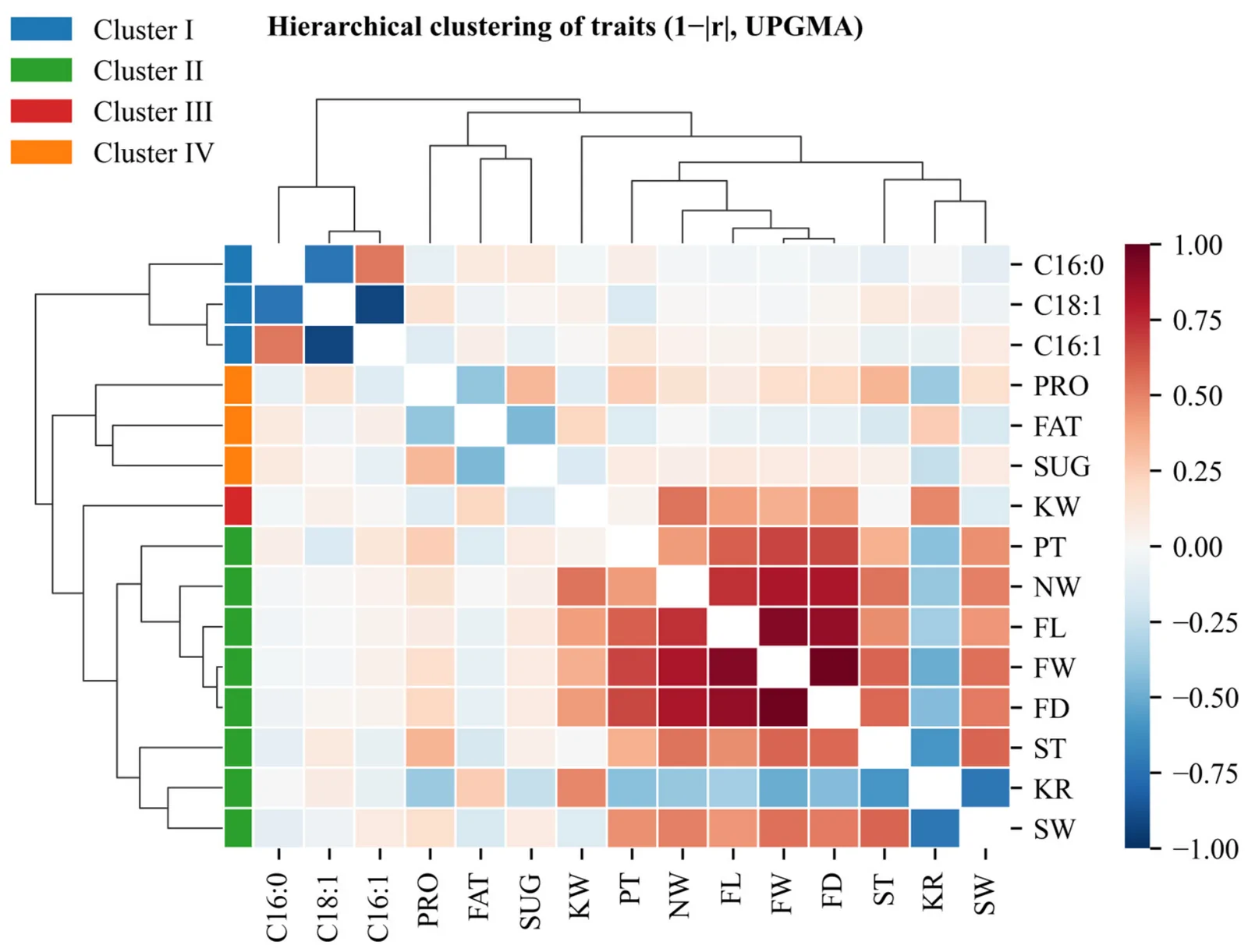
Correlation heatmap and R clustering dendrogram of major fatty acids and fruit traits. Note: Palmitic acid (C16:0); oleic acid (C18:1); palmitoleic acid (C16:1); crude protein content (PRO); crude fat content (FAT); total soluble sugar content (SUG); single dry kernel weight (KW); peel thickness (PT); single dry nut weight (NW); fruit longitudinal diameter (FL); fruit fresh weight (FW); fruit equatorial diameter (FD); shell thickness at the hilum (ST); kernel recovery (KR); shell wall thickness at sides (SW).

Correlation analysis was performed on the three major fatty acids, nine fruit traits, and three kernel quality traits, and hierarchical clustering was conducted using 1 − |r| as the distance measure with average linkage to explore the relationships among variables. The results showed that the 15 traits were divided into four clusters (Figure 2). Cluster I comprised C16:0, C18:1, and C16:1, among which the correlations were highly significant (*p* < 0.001); C18:1 was strongly negatively correlated with C16:0 (r = −0.733) and C16:1 (r = −0.921), whereas C16:1 was strongly positively correlated with C16:0 (r = 0.534), suggesting possible metabolic competition among these fatty acids [2,22]. Cluster II comprised fruit size and peel/shell thickness traits (KR, FW, NW, FL, FD, SW, ST, and PT), which were highly significantly and moderately to strongly correlated with one another (|r| = 0.345–0.970, *p* < 0.001); KR was negatively correlated with the other traits in this cluster, whereas the other traits were positively correlated with one another, indicating that larger fruits had thicker peel and shells and lower kernel recovery. Cluster III comprised KW alone, whose correlations were generally consistent with those of Cluster II, but KW was not significantly correlated with PT, ST, or SW (|r| = 0.006–0.133), indicating that KW is independent of peel and shell thickness traits. Cluster IV comprised FAT, PRO, and SUG, the kernel quality traits, which were highly significantly correlated with one another (*p* < 0.001); FAT was weakly to moderately negatively correlated with PRO and SUG (r = −0.392 and −0.449, respectively), and PRO was weakly positively correlated with SUG (r = 0.336).

### 3.3. Principal Component Analysis of Fatty Acids and Fruit Traits

To explore the variation structure of the 139 accessions, PCA was performed on 15 standardized traits (three major fatty acids, nine fruit traits, and three kernel quality traits), and the eigenvalue of each principal component represents the amount of variance it explains. According to the Kaiser criterion, principal components with eigenvalues greater than 1 were retained; four principal components were extracted (Table 3), with a cumulative variance contribution of 76.89%, which explains the main variation of the accessions.

The first principal component (PC1) had the largest variance contribution (35.88%), with fruit fresh weight, fruit equatorial diameter, fruit longitudinal diameter, and single nut weight as the main contributing variables, representing the variation in fruit weight and size. PC2 accounted for 17.15% of the total variance, in which C18:1 (positive loading 0.58), C16:1 (negative loading −0.55), and C16:0 (negative loading −0.48) had the largest absolute loadings, and the signs of the loadings were consistent with the correlation relationships, reflecting the metabolic competition between C18:1 and C16:1. PC3 and PC4 accounted for 15.26% and 8.61% of the variance, respectively; single dry kernel weight and kernel recovery were the main contributing variables of PC3, and soluble sugar (positive loading) and crude fat content (negative loading) were the main contributors of PC4, comprehensively reflecting the kernel quality of the accessions.

**Figure 3:**
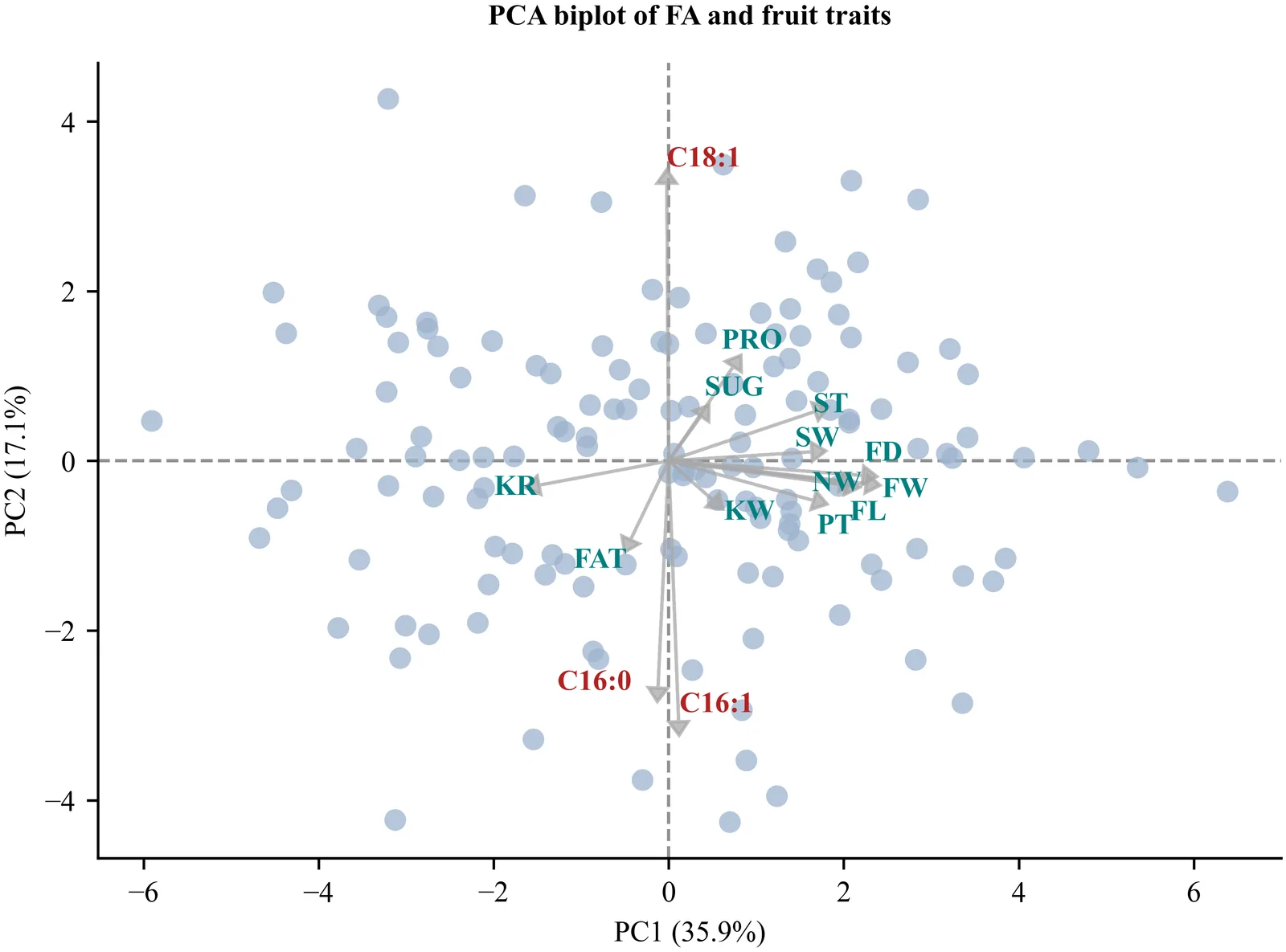
PCA biplot of the 139 macadamia accessions. Note: Palmitic acid (C16:0); oleic acid (C18:1); palmitoleic acid (C16:1); crude protein content (PRO); crude fat content (FAT); total soluble sugar content (SUG); single dry kernel weight (KW); peel thickness (PT); single dry nut weight (NW); fruit longitudinal diameter (FL); fruit fresh weight (FW); fruit equatorial diameter (FD); shell thickness at the hilum (ST); kernel recovery (KR); shell wall thickness at sides (SW).

A genotype × trait biplot was constructed using the first two principal components with the largest variance contributions (Figure 3) to display the distribution of the 139 accessions. The biplot covered three major fatty acids, nine fruit traits, and three kernel quality traits; the distribution along PC1 reflected the differences among accessions in fruit weight and size, whereas the distribution along PC2 reflected the differences in the composition of the three major fatty acids. The nearly orthogonal arrangement of fruit trait and fatty acid composition vectors is consistent with the independence of fruit traits and fatty acid contents in the correlation analysis, and the 139 accessions were scattered across the four quadrants, indicating a certain degree of variation among accessions, with 38 accessions distributed in Q4, suggesting the likely existence of accessions with both relatively high C16:1 and desirable fruit traits.

### 3.4. Selection of Candidate Accessions Based on Entropy-Weighted TOPSIS

Among the 22 fatty acids detected, only three accounted for more than 5% of the total fatty acids (C18:1, 74.75%; C16:1, 10.66%; C16:0, 5.76%), whereas the remaining 19 acids together contributed 8.8%, most of them below 1% (Table 1). In the R clustering, C16:0, C16:1 and C18:1 were assigned to the same cluster (Cluster I, Figure 2). Because C16:1 and C18:1 were strongly negatively correlated (r = −0.921), including both would duplicate the weighting of the same information, and C18:1 also showed the smallest variation (CV = 5.3%) and therefore the lowest discriminating power; C16:1 was thus retained as the representative of this cluster, whereas C16:0 and C18:1 were not included separately because of their overlap with C16:1, and the remaining fatty acids were excluded for their very low contents. Following the same clustering result (Figure 2), one to two traits were selected from each cluster, giving a final set of five benefit-type indicators: C16:1, fruit fresh weight, kernel recovery, single dry kernel weight and crude fat content, including the fruit traits that determine market value [27], which were used in the TOPSIS analysis. The entropy weight method was used to calculate the weights of the indicators, and TOPSIS was then used to calculate the positive and negative Euclidean distances of the 139 accessions to the ideal solutions; finally, the accessions were ranked in descending order by relative closeness (C) to identify accessions with both high C16:1 content and desirable fruit traits. The results showed (Table 4) that among the five indicators, C16:1 had the largest weight and crude fat had the smallest weight; the sum of the weights of the three fruit traits was 0.51, which is consistent with the coefficients of variation in the descriptive statistics above and in line with production practice. The TOPSIS results (Table 5) showed that the C16:1 content of the top 10 accessions was higher than the population mean (10.658%, Table 1), the complete ranking of all 139 accessions is provided in Table S2. According to the elite cultivar selection standards (kernel recovery > 32%, single dry kernel weight > 2 g), accessions No. 53, 137, 20, 9, and 39 were preliminarily selected. These five accessions originated from Australia (53), Yunnan, China (137), and Hawaii (20, 9, and 39), indicating that elite materials combining high C16:1 content with desirable fruit traits exist in different origins.

## 4. Discussion

The results of this study showed that MUFA was the predominant fatty acid class in the kernels of the 139 accessions, with an average content of 88.275%, which is higher than the 72–80% [7] and >70% [8] reported in the literature. The average C18:1 content was 74.745%, higher than the reported 57–67% [2], 40–51% [7], and 51–64% [8]. The average C16:1 content was 10.658%, lower than the 15–22% [11], 14–24% [2], and 24–36% [7] reported in the literature, but within the 10–21% range reported for cold-pressed macadamia oil [8]. The ranking pattern of the major fatty acids (C18:1 > C16:1 > C16:0) was consistent with most previous studies [2,7]. This comparison is intended only to indicate where our values lie relative to those reported in the literature. Analytical methods differ among studies, and the fatty acid composition of macadamia oil has been reported to be affected by genetic, environmental and cultivation factors (rainfall, temperature, water stress, and nitrogen fertilizer) as well as extraction methods [7], and climatic conditions can also significantly alter the saturated and unsaturated composition of the oil [19]. Several factors may contribute to the relatively low mean C16:1 content of this population relative to literature values: (1) the germplasm composition is dominated by Chinese seedling selections (46.0%), with Hawaiian and Australian commercial cultivars accounting for relatively small proportions, and systematic differences in fatty acid composition may exist among accessions of different origins; (2) GC-MS was used in this study to directly quantify fresh kernel samples, which differs from the gas chromatography–cold-pressed oil analysis commonly used in the literature in terms of sample pretreatment and detection methods; (3) the tested materials were grown in the northern tropical climate zone of Jinghong, Yunnan, which may differ from the production environments of the literature studies. In addition, the difference in fatty acid composition of macadamia before and after roasting is minimal [6], indicating that processing methods have limited effects on its fatty acid composition.

The results of this study showed that the coefficient of variation of C16:1 among accessions was 27.7%, much higher than 5.3% for C18:1, whereas the RSD of C16:1 in QC samples was only 2.3%; the inter-accession variation of C16:1 was approximately 12 times the measurement error, indicating that the differences in C16:1 content among accessions mainly originate from inter-accession differences rather than measurement error. The frequency distribution results showed that C16:1 was discretely distributed with the peak spanning −0.1 SD to +1 SD, whereas C18:1 was concentrated near the mean. In terms of content range, C16:1 ranged from 2.783% to 19.047%, and C18:1 from 64.459% to 85.779%; the wide distribution and variability of C16:1 contrasted sharply with the concentrated distribution of C18:1. Previous studies have also shown that the variation in fatty acid profiles is largely genetically determined [2]; the analysis of fatty acid profiles of 197 accessions also showed extensive variation among accessions [22]; and studies on olive oil showed that genetic factors explained 50–90% of the variation in fatty acid profiles, whereas the contribution of interannual factors was only about 4–18% [19]. These results are consistent with those of the present study, indicating that the inter-accession differences in C16:1 have a stable genetic basis, that screening for high-C16:1 materials among the 139 accessions is feasible, and that C16:1 content shows promise for genetic improvement.

Correlation analysis revealed that the three major fatty acids were significantly inter-correlated, suggesting possible metabolic competition among them. In the fatty acid biosynthesis pathway, C16:0 is desaturated to C16:1 and C18:0 is desaturated to C18:1; the two share the same desaturation pathway, and the elongation of C16:0 to C18:0 competes with the desaturation of C16:0 for the same substrate [2]. Studies on macadamia showed that the partitioning ratio between C16 desaturation and C16 elongation was strongly negatively correlated (r^2^ = 92%), further confirming that the two compete for the same substrate (ACP-C16:0) [2]. Based on the analysis of fatty acid interactions in 197 accessions, Hu et al. proposed two possible desaturation pathway hypotheses, namely the C16:0-specific desaturase hypothesis and the hypothesis of low substrate specificity of the C18:0 desaturase for C16:0 [22]; the strong negative correlation between C16:1 and C18:1 in this study is consistent with the above pathway competition model. The high content of C16:1 in macadamia may originate from a divergent acyl-ACP desaturase with high substrate specificity for 16:0-ACP [11]. In thioesterase studies, *Macadamia tetraphylla* fatty acyl–ACP thioesterase A (MtFatA) showed high catalytic efficiency toward 16:1-ACP, whereas MtFatB preferred 16:0-ACP, indicating that the substrate specificity of thioesterases is involved in regulating the accumulation of n-7 fatty acids [28]. In addition, the type I diacylglycerol acyltransferase MtDGAT1 prefers monounsaturated fatty acid substrates in heterologous expression [29]; ketoacyl-ACP synthase (KAS) and stearoyl-ACP desaturase (SAD/FAB2) are key enzymes in fatty acid elongation and desaturation [30], and chromosome-scale genome analysis identified 187 genes encoding 33 key enzymes of lipid synthesis [31]. Fatty acid accumulation is mainly concentrated in the middle stage of fruit development (80–125 d after flowering) [32], and combined transcriptome and lipidome analysis identified candidate lipid synthesis genes such as glycerol-3-phosphate acyltransferase and 3-ketoacyl-ACP reductase [33]. The strong negative correlation between C16:1 and C18:1 in this study is consistent with the above metabolic competition hypothesis: when the proportion of substrate flowing to C16:1 in the desaturation pathway increases, the proportion flowing to C18:1 correspondingly decreases, and vice versa.

In the correlation analysis, the direct correlations between the three major fatty acids and fruit traits were all non-significant; in the PCA, fruit-size-related traits were mainly distributed on PC1 (variance contribution 35.88%), while the three fatty acids were concentrated on PC2 (17.15%), and the fruit trait vectors and fatty acid composition vectors were nearly orthogonal in the biplot. This indicates that fatty acid content and fruit traits are generally independent of each other. Owing to the highly significant strong negative correlation between C16:1 and C18:1 (r = −0.921, *p* < 0.001), partial correlation analysis controlling for C18:1 showed that C16:1 was weakly but significantly positively correlated with single dry kernel weight (r = 0.170, *p* < 0.05), but not significantly correlated with kernel recovery (r = 0.000) or crude fat content (r = 0.044). This indicates that accessions with high C16:1 content have slightly higher single kernel weight, while kernel recovery and crude fat content are not affected, so that selection for high C16:1 can be combined with the improvement of fruit traits.

Based on the relationships between fatty acids and fruit traits described above and on the R-clustering results (Figure 2), one to two traits was retained from each of the four clusters, giving C16:1, FW, KR, KW and FAT as the final evaluation indicators. Entropy-weighted TOPSIS was then applied together with the thresholds of KR > 32% and KW > 2.0 g, and five elite accessions were selected. Here, “elite” denotes accessions that combine high C16:1 with favorable economic fruit traits. C16:1 reflects nutritional and functional quality, because this fatty acid is uncommon in the plant kingdom and macadamia kernels can serve as a dietary source of it [11]. FW, KR and KW reflect yield potential, as they directly determine growers’ income [27] and the market price of macadamia kernels [1]. Crude fat content reflects oil quality, since macadamia kernels contain more than 70% oil and a high oil content is associated with superior nutritional quality [4]. All of the high-C16:1 accessions selected here had kernel recoveries above 32%, indicating that fatty acid composition can be improved without compromising the traits that underpin grower income.

The data in this study were derived from a single year and a single location (the same germplasm repository and the same production season), and interannual effects have not been evaluated. As noted above, although interannual effects are relatively small, climatic conditions can still significantly alter the saturated and unsaturated fatty acid composition of the oil [19]. On the other hand, all accessions in this study were planted and sampled in the same germplasm repository under the same cultivation and management conditions during the same period, which eliminated the interference of differences in location, soil, and management measures on the comparison among accessions; therefore, the detected inter-accession variation in C16:1 (CV = 27.7%) mainly reflects genetic differences. The interannual stability of the candidate accessions still needs to be verified with multi-year data, which is the focus of follow-up work.

In the future, the five candidate accessions can be evaluated in multiple years and locations to assess the stability and environmental adaptability of their fatty acid composition [19]; using the genomic resources of macadamia [34,35] and the genetic diversity of wild germplasm [21], the genes related to C16:1 content can be identified by genome-wide association study (GWAS), and the breeding cycle can be shortened by combining marker-assisted selection [36], thereby supporting the breeding of macadamia cultivars specifically for high-C16:1 oil.

## 5. Conclusions

In conclusion, this study characterized the kernel fatty acid composition of 139 macadamia accessions and examined how the major fatty acids relate to one another and to fruit traits. The major fatty acids ranked as C18:1 > C16:1 > C16:0, with C16:1 varying considerably among accessions while C18:1 remained stable; their strong negative correlation points to a biosynthetic trade-off in which each is produced at the expense of the other. Fatty acid composition was independent of fruit traits, showing that high C16:1 and favorable fruit characteristics can be improved simultaneously. On this basis, five accessions (No. 53, 137, 20, 9 and 39) combining high C16:1 with desirable fruit traits were selected by entropy-weighted TOPSIS as candidate parents for cultivar development. These results clarify how the major fatty acids of macadamia are interrelated and provide both breeding material and a practical screening framework for improving oil nutritional quality. Future work should verify the stability of these profiles across years and locations, and then combine genome-wide association studies with marker-assisted selection to introgress favorable C16:1 alleles into elite cultivars.

## Figures and Tables

**Table 1:** Descriptive statistics of relative proportions of individual fatty acids in the kernels of the 139 macadamia accessions (%).

FA	Mean ± SD	Min	Max	CV (%)	QC-RSD
C6-0	0.032 ± 0.019	0.007	0.117	58.4	9.99
C8-0	0.019 ± 0.007	0.005	0.037	40.1	7.97
C9-0	0.016 ± 0.016	0.000	0.067	99.0	7.65
C10-0	0.021 ± 0.007	0.007	0.043	31.7	1.79
C11-0	0.015 ± 0.004	0.006	0.029	28.6	3.83
C12-0	0.049 ± 0.021	0.012	0.122	43.2	1.41
C14-0	0.395 ± 0.174	0.097	0.921	44.1	1.58
C15-0	0.008 ± 0.004	0.003	0.026	46.0	3.33
C16-0	5.757 ± 0.851	3.314	8.942	14.8	2.01
C17-0	0.015 ± 0.005	0.007	0.034	29.9	13.1
C18-0	0.744 ± 0.236	0.383	1.453	31.7	1.24
C20-0	0.439 ± 0.185	0.220	1.473	42.3	1.10
C22-0	0.100 ± 0.053	0.033	0.423	53.1	3.54
C24-0	0.042 ± 0.030	0.006	0.208	71.2	1.03
SFA	7.652 ± 1.215	5.130	11.755	15.9	
C16-1n7c	10.658 ± 2.950	2.783	19.047	27.7	2.30
C18-1n9c	74.745 ± 3.962	64.459	85.779	5.3	3.89
C18-1n9t	2.348 ± 0.602	1.286	5.355	25.6	3.74
C20-1n9c	0.523 ± 0.199	0.233	2.118	38.1	0.26
MUFA	88.275 ± 1.455	83.858	91.02	1.6	
C18-2n6t	1.751 ± 0.307	1.100	2.289	17.5	1.65
C18-2n6c	1.748 ± 0.679	0.669	4.322	38.8	1.21
C18-3n3c	0.564 ± 0.092	0.341	0.836	16.4	1.24
PUFA	4.062 ± 0.855	2.109	7.236	21.1	

Note: Relative FA proportions for all accessions are given in Table S3.

**Table 2:** Analysis of variance of fruit traits and kernel quality traits in the 139 macadamia accessions.

Trait	Mean ± SD	Min	Max	CV (%)	F Value	η^2^
Fruit fresh weight (g)	19.84 ± 3.49 ***	12.70	29.20	17.6	54.89	0.74
Fruit equatorial diameter (mm)	31.64 ± 1.86 ***	27.17	36.40	5.9	45.16	0.70
Fruit longitudinal diameter (mm)	33.10 ± 1.93 ***	27.69	37.82	5.8	44.13	0.69
Peel thickness (mm)	4.12 ± 0.51 ***	2.77	5.60	12.4	33.49	0.63
Single dry kernel weight (g)	2.35 ± 0.41 ***	1.26	3.53	17.4	10.70	0.83
Kernel recovery (%)	32.87 ± 4.86 ***	16.90	46.41	14.8	28.45	0.93
Single dry nut weight (g)	7.22 ± 1.23 ***	4.11	11.30	17.1	8.66	0.80
Shell thickness at the hilum (mm)	5.42 ± 1.04 ***	2.47	8.26	19.2	20.96	0.69
Shell wall thickness at sides (mm)	2.43 ± 0.4 ***	1.54	3.85	16.5	30.41	0.61
Crude protein content (%)	9.66 ± 0.87 ***	7.48	11.48	9.0	25.2	0.92
Crude fat content (%)	74.75 ± 1.37 ***	71.67	77.57	1.8	5.20	0.71
Total soluble sugar content (%)	3.32 ± 0.77 ***	1.56	5.02	23.1	6.70	0.76

Note: In the permutation ANOVA, accession labels were randomly permuted 9999 times, with the F statistic recomputed for each permutation, to generate an empirical null distribution of F. The observed F value was then compared with this null distribution to obtain the permutation *p*-value, which requires no assumptions of normality or homogeneity of variance. The significance markers “***” in this table are based on these permutation *p*-values (<0.001). The observed F values are reported because F is the test statistic used in the permutation approach. η^2^ = SS_between_/(SS_between_ + SS_within_), where SS_between_ is the sum of squares between accessions and SS_within_ is the residual (within accession) sum of squares, η^2^ quantifies the proportion of the total variance in a trait that is attributable to differences among accessions. The raw FAT, PRO, and SUG data for all replicates of all accessions are given in Table S4, and the corresponding spectral data (2 nm resolution) are given in Table S5.

**Table 3:** Eigenvectors, eigenvalues, and individual and cumulative percentages of variation explained by the first five principal components (PCs) of 139 macadamia accessions.

Trait	PC1	PC2	PC3	PC4	PC5
Oleic acid (C18:1)	−0.0039	0.5817	0.1977	−0.0345	−0.0433
Palmitoleic acid (C16:1)	0.0203	−0.5481	−0.1496	−0.0023	0.0963
Palmitic acid (C16:0)	−0.0220	−0.4808	−0.1550	0.1874	0.1289
Single dry kernel weight	0.1052	−0.0965	0.5346	0.3412	0.2323
Kernel recovery	−0.2776	−0.0537	0.3964	0.3016	0.0048
Single dry nut weight	0.3589	−0.0455	0.2056	0.0579	0.1957
Fruit fresh weight	0.4094	−0.0488	0.1308	0.0298	−0.1183
Fruit equatorial diameter	0.4032	−0.0313	0.1542	0.0720	−0.0559
Fruit longitudinal diameter	0.3700	−0.0526	0.1889	0.1017	−0.2258
Peel thickness	0.3068	−0.0865	−0.0672	0.0114	−0.3488
Shell thickness at the hilum	0.3089	0.1074	−0.1024	−0.2037	0.4461
Shell wall thickness at sides	0.3043	0.0199	−0.1924	−0.3564	−0.0782
Crude protein content	0.1400	0.2109	−0.2901	0.2957	0.6294
Crude fat content	−0.0853	−0.1822	0.3500	−0.4141	0.1598
Total soluble sugar content	0.0780	0.1114	−0.3067	0.5558	−0.2518
Eigenvalue	5.420	2.591	2.305	1.302	0.725
Variability (%)	35.876	17.148	15.256	8.614	4.797
Cumulative (%)	35.876	53.024	68.279	76.894	81.690

**Table 4:** Weights of the selection indicators determined by the entropy weight method.

Indicator	Entropy Value	Difference Coefficient	Weight
Palmitoleic acid	0.9921	0.0079	0.4844
Fruit fresh weight	0.9969	0.0031	0.1903
Kernel recovery	0.9978	0.0022	0.1363
Single dry kernel weight	0.9969	0.0031	0.1870
Crude fat content	1.0000	0.0000	0.0021

**Table 5:** TOPSIS ranking results (top 10).

No.	Origin	C16:1	FW	KR	KW	FAT	D+	D−	C	Rank
**53**	**Australia**	**19.05**	**21.40 ± 2.03**	**34.19 ± 0.41**	**2.90 ± 0.03**	**75.63 ± 1.39**	**0.0087**	**0.0621**	**0.8776**	**1**
61	Hawaii	16.72	20.02 ± 1.73	30.92 ± 0.22	2.51 ± 0.13	74.43 ± 0.75	0.0143	0.053	0.7878	2
19	Hawaii	18.40	16.16 ± 1.67	30.25 ± 1.46	1.70 ± 0.13	74.70 ± 0.89	0.0172	0.0583	0.7724	3
**137**	**China**	**16.44**	**19.78 ± 1.41**	**33.05 ± 0.35**	**2.33 ± 0.31**	**75.37 ± 0.82**	**0.0154**	**0.0518**	**0.7712**	**4**
**20**	**Hawaii**	**15.95**	**20.59 ± 1.49**	**34.19 ± 1.42**	**2.63 ± 0.18**	**76.72 ± 1.93**	**0.0153**	**0.0505**	**0.7675**	**5**
96	Australia	15.61	27.18 ± 3.01	28.73 ± 0.59	2.53 ± 0.39	76.35 ± 0.56	0.0157	0.0500	0.7605	6
**9**	**Hawaii**	**16.52**	**18.35 ± 1.92**	**32.50 ± 2.39**	**2.03 ± 0.26**	**74.01 ± 2.84**	**0.0169**	**0.0518**	**0.7534**	**7**
13	Hawaii	15.77	18.96 ± 1.43	31.99 ± 0.76	2.17 ± 0.04	74.56 ± 0.19	0.0179	0.0492	0.7327	8
**39**	**Hawaii**	**15.21**	**16.96 ± 1.62**	**39.67 ± 0.42**	**2.85 ± 0.04**	**74.85 ± 1.05**	**0.0181**	**0.0481**	**0.7272**	**9**
110	China	15.71	20.20 ± 1.73	28.27 ± 0.99	2.00 ± 0.08	74.86 ± 0.69	0.0187	0.0488	0.7235	10

Note: Palmitoleic acid (C16:1); fruit fresh weight (FW); kernel recovery (KR); single dry kernel weight (KW); crude fat content (FAT); positive Euclidean distances to the ideal solutions (D+); negative Euclidean distances to the ideal solutions (D−); relative closeness (C). Values are mean ± SD (*n* = 20 for FW; 3 for KR, KW and FAT); C16:1 was determined on a single composite sample per accession (*n* = 1), so no SD is given. Bold rows indicate accessions that also met KR > 32% and KW > 2 g.

## Data Availability

The original contributions presented in the study are included in the article, further inquiries can be directed to the corresponding author.

## Supplementary Materials

The following supporting information can be downloaded at https://www.mdpi.com/article/10.3390/foods15213877/s1, Table S1: List of the 139 macadamia accessions used in this study; Table S2: Complete TOPSIS ranking of the 139 macadamia accessions based on the entropy weight method; Table S3: FA information of all accessions; Table S4: Raw data of FAT, PRO, and SUG for all germplasm replicates; Table S5: Raw NIRS spectral data (2 nm resolution) for all germplasm replicates.
