# Supplementary material for: Fatty Acid Variation in Macadamia Kernels: Association of Palmitoleic Acid with Fruit Traits and Selection of Multi-Attribute Germplasm
Source: Foods. 2026 Oct 29;15(21):3877. doi: 10.3390/foods15213877 (PMC13763349; doi:10.3390/foods15213877)
Supplement: Supplementary file 1 [file foods-15-03877-s001.zip › Table S1 139 macadamia accessions.pdf]

Table S1. List of the 139 macadamia accessions used in this study.

No.	Accession name	Origin	Type
2	Guang 11	China	Chinese cultivar
3	HAES246	Hawaii	Introduced cultivar
4	HAES294	Hawaii	Introduced cultivar
5	HAES333	Hawaii	Introduced cultivar
6	HAES344	Hawaii	Introduced cultivar
7	HAES508	Hawaii	Introduced cultivar
8	HAES660	Hawaii	Introduced cultivar
9	HAES695	Hawaii	Introduced cultivar
10	HAES741	Hawaii	Introduced cultivar
12	HAES762	Hawaii	Introduced cultivar
13	HAES772	Hawaii	Introduced cultivar
15	HAES780	Hawaii	Introduced cultivar
16	HAES783	Hawaii	Introduced cultivar
17	HAES788	Hawaii	Introduced cultivar
18	HAES789	Hawaii	Introduced cultivar
19	HAES790	Hawaii	Introduced cultivar
20	HAES792	Hawaii	Introduced cultivar
21	HAES800	Hawaii	Introduced cultivar
22	HAES812	Hawaii	Introduced cultivar
23	HAES814	Hawaii	Introduced cultivar
24	HAES815	Hawaii	Introduced cultivar
25	HAES816	Hawaii	Introduced cultivar
26	HAES819	Hawaii	Introduced cultivar
29	HAES828	Hawaii	Introduced cultivar
32	HAES842	Hawaii	Introduced cultivar
34	HAES849	Hawaii	Introduced cultivar
35	HAES851	Hawaii	Introduced cultivar
36	HAES854	Hawaii	Introduced cultivar
39	HAES863	Hawaii	Introduced cultivar
41	HAES900	Hawaii	Introduced cultivar
42	HAES904	Hawaii	Introduced cultivar
44	HAES915	Hawaii	Introduced cultivar
45	HAES918	Hawaii	Introduced cultivar
46	HAES932	Hawaii	Introduced cultivar
50	D	Australia	Introduced cultivar
53	T	Australia	Introduced cultivar
60	H3	Hawaii	Introduced cultivar
61	AH-H	Hawaii	Introduced cultivar
62	HW1	Hawaii	Introduced cultivar
63	Nanya 1	China	Chinese cultivar
65	800 TCS	China	Tissue-cultured seedling
66	Guire 1	China	Chinese cultivar
67	HY01	Hawaii	Introduced cultivar
68	AY02	Australia	Introduced cultivar
69	AY03	Australia	Introduced cultivar
70	AY04	Australia	Introduced cultivar
73	AY06	Australia	Introduced cultivar
74	AY07	Australia	Introduced cultivar
75	AY08	Australia	Introduced cultivar
76	AY09	Australia	Introduced cultivar
77	AY10	Australia	Introduced cultivar
79	IY01	Australia	Introduced cultivar
80	AY12	Australia	Introduced cultivar
81	AY13	Australia	Introduced cultivar
82	AY14	Australia	Introduced cultivar
83	AY15	Australia	Introduced cultivar
85	AY16	Australia	Introduced cultivar

86	AY17	Australia	Introduced cultivar
87	AY18	Australia	Introduced cultivar
88	AY19	Australia	Introduced cultivar
89	HY02	Australia	Introduced cultivar
90	AY20	Australia	Introduced cultivar
91	AY21	Australia	Introduced cultivar
92	Huanan 788	China	Seedling selection
95	C-H	Australia	*M. tetraphylla*
96	AY24	Australia	Introduced cultivar
97	AY23	Australia	Introduced cultivar
98	Nanya 2	China	Chinese cultivar
100	VN01	Australia	Introduced cultivar
101	Cold resistant	China	Seedling selection
102	JH05	China	Seedling selection
103	JH07	China	Seedling selection
104	JH08	China	Seedling selection
105	JH09	China	Seedling selection
108	JH13	China	Seedling selection
109	JH14	China	Seedling selection
110	JH15	China	Seedling selection
112	JH18	China	Seedling selection
113	JH19	China	Seedling selection
114	JH21	China	Seedling selection
115	JH22	China	Seedling selection
116	JH23	China	Seedling selection
117	JH24	China	Seedling selection
119	JH26	China	Seedling selection
120	JH27	China	Seedling selection
121	JH28	China	Seedling selection
123	JH31	China	Seedling selection
125	JH34	China	Seedling selection
126	JH35	China	Seedling selection
132	JH41	China	Seedling selection
133	JH42	China	Seedling selection
134	JH44	China	Seedling selection
137	JH47	China	Seedling selection
138	JH48	China	Seedling selection
139	JH50	China	Seedling selection
141	JH52	China	Seedling selection
142	JH53	China	Seedling selection
143	JH54	China	Seedling selection
146	JH57	China	Seedling selection
147	JH58	China	Seedling selection
154	JH65	China	Seedling selection
156	JH67	China	Seedling selection
157	JH69	China	Seedling selection
158	JH70	China	Seedling selection
159	JH71	China	Seedling selection
161	JH73	China	Seedling selection
162	JH74	China	Seedling selection
163	JH75	China	Seedling selection
164	JH76	China	Seedling selection
165	JH77	China	Seedling selection
166	JH78	China	Seedling selection
169	JHG1	China	Seedling selection
170	JHG2	China	Seedling selection
173	JHG6	China	Seedling selection
174	Lei1	China	Seedling selection
177	Lei4	China	Seedling selection
178	Lei5	China	Seedling selection

179	Lei6	China	Seedling selection
184	MD1	China	Seedling selection
186	MD3	China	Seedling selection
187	MD4	China	Seedling selection
189	Ke2	China	Seedling selection
190	Ke3	China	Seedling selection
194	Bai2	China	Seedling selection
200	Yukun1	China	Seedling selection
202	DS1	China	Chinese cultivar
205	YXC1	China	Seedling selection
206	YXC3	China	Seedling selection
207	YXC4	China	Seedling selection
208	MT5	China	Seedling selection
209	MT20	China	Seedling selection
211	WD31	China	Seedling selection
212	WD37	China	Seedling selection
215	MT10	China	Seedling selection
218	WD34	China	Seedling selection
223	HY03	Hawaii	Introduced cultivar
234	JH3	China	Seedling selection
242	38 seedling 2	China	Seedling selection
251	C 4	Australia	*M. tetraphylla*

Note: All accessions belong to the genus *Macadamia*. Species names are given only where the species identity has been confirmed; for the remaining accessions, species assignment is under further investigation.
