# Supplementary material for: Fatty Acid Variation in Macadamia Kernels: Association of Palmitoleic Acid with Fruit Traits and Selection of Multi-Attribute Germplasm
Source: Foods. 2026 Oct 29;15(21):3877. doi: 10.3390/foods15213877 (PMC13763349; doi:10.3390/foods15213877)
Supplement: Supplementary file 1 [file foods-15-03877-s001.zip › Table S2 TOPSIS ranking 139 accessions.pdf]

Table S2. Complete TOPSIS ranking of the 139 macadamia accessions based on the entropy weight method.

No.	Accession name	Origin	C16:1/%	FW/g	KR/%	KW/g	FAT/%	D+	D-	C	Rank
53	T	Australia	19.05	21.4	34.19	2.9	75.63	0.0087	0.0621	0.8776	1
61	AH-H	Hawaii	16.72	20.02	30.92	2.51	74.43	0.0143	0.053	0.7878	2
19	HAES790	Hawaii	18.4	16.16	30.25	1.7	74.7	0.0172	0.0583	0.7724	3
137	JH47	China	16.44	19.78	33.05	2.33	75.37	0.0154	0.0518	0.7712	4
20	HAES792	Hawaii	15.95	20.59	34.19	2.63	76.72	0.0153	0.0505	0.7675	5
96	AY24	Australia	15.61	27.18	28.73	2.53	76.35	0.0157	0.05	0.7605	6
9	HAES695	Hawaii	16.52	18.35	32.5	2.03	74.01	0.0169	0.0518	0.7534	7
13	HAES772	Hawaii	15.77	18.96	31.99	2.17	74.56	0.0179	0.0492	0.7327	8
39	HAES863	Hawaii	15.21	16.96	39.67	2.85	74.85	0.0181	0.0481	0.7272	9
110	JH15	China	15.71	20.2	28.27	2	74.86	0.0187	0.0488	0.7235	10
234	JH3	China	14.87	25.76	29.88	2.44	76.21	0.0183	0.047	0.7198	11
68	AY02	Australia	15.01	17.72	37.57	2.72	73.12	0.0187	0.0472	0.7166	12
4	HAES294	Hawaii	14.61	23.35	33.53	2.83	77.44	0.0183	0.0463	0.7165	13
18	HAES789	Hawaii	15.05	15.41	37.92	2.36	76.57	0.0203	0.0468	0.6974	14
46	HAES932	Hawaii	14.27	20.07	34.34	2.5	75.72	0.0208	0.0443	0.6805	15
69	AY03	Australia	14.59	15.07	41.58	2.32	74.33	0.0217	0.0453	0.6762	16
97	AY23	Australia	14.23	21.58	31.67	2.23	75.88	0.0214	0.0439	0.6719	17
170	JHG2	China	13.63	22.16	33.13	2.86	76.53	0.0219	0.0427	0.6614	18
154	JH65	China	13.74	23.84	28.33	2.63	73.57	0.022	0.0428	0.6608	19
25	HAES816	Hawaii	13.49	17.22	45.64	3.31	74.72	0.0228	0.0434	0.6554	20
132	JH41	China	13.93	18.99	26.68	1.76	74.63	0.0248	0.042	0.6287	21
211	WD31	China	12.49	25.06	38.29	3.53	75.8	0.0247	0.041	0.6237	22
115	JH22	China	13.16	20.42	34.88	2.29	75.29	0.0247	0.0401	0.6188	23
34	HAES849	Hawaii	12.68	19.97	42.66	3.17	74.83	0.025	0.0403	0.6178	24
141	JH52	China	12.8	22.55	33.22	2.83	74.97	0.0247	0.0399	0.6177	25
113	JH19	China	12.92	22.91	32.72	2.51	75.57	0.0247	0.0398	0.6169	26
147	JH58	China	13.12	21.82	26.23	2.21	74.49	0.0254	0.0397	0.6097	27
142	JH53	China	13.01	19.94	31.68	2.41	73.78	0.0253	0.0395	0.6095	28
119	JH26	China	12.68	23.19	30.31	2.74	73.43	0.0253	0.0393	0.6077	29
5	HAES333	Hawaii	13.1	18.68	31.63	2.22	74.4	0.0257	0.0395	0.6057	30
10	HAES741	Hawaii	13.17	16.21	35.85	2.18	77.27	0.026	0.0397	0.6041	31
89	HY02	Hawaii	13.2	13.47	39.3	2.38	75.3	0.0264	0.0402	0.6039	32
163	JH75	China	12.63	22.08	30.38	2.72	75.12	0.0257	0.0389	0.6017	33
24	HAES815	Hawaii	12.86	18.49	36.35	2.4	73.73	0.0259	0.0391	0.6014	34
95	C-H	Australia	13.5	26.34	16.9	1.26	71.67	0.0276	0.0413	0.5989	35
134	JH44	China	12.6	20.64	33.29	2.27	73.82	0.0267	0.0381	0.5877	36
109	JH14	China	12.75	21.08	27.03	2.03	76.7	0.0271	0.0381	0.5843	37
105	JH09	China	12.48	20.97	33.62	2.34	73.63	0.0269	0.0378	0.5842	38
35	HAES851	Hawaii	12.84	16.2	35.15	2.08	75.57	0.0274	0.0384	0.5838	39
166	JH78	China	12.27	27.86	27.74	2.32	75.83	0.0272	0.0381	0.5834	40
92	Huanan 788	China	13.26	15.21	28.03	1.64	74.78	0.0281	0.0393	0.5831	41
207	YXC4	China	12.63	17.8	29.56	2.3	75.8	0.0274	0.0377	0.5789	42

73	AY06	Australia	11.95	21.72	34.34	2.86	75.28	0.0277	0.0369	0.5712	43
6	HAES344	Hawaii	12.31	19.68	30.1	2.42	75.95	0.0278	0.037	0.571	44
114	JH21	China	12.19	26.91	21.54	1.96	73.64	0.0289	0.0371	0.5616	45
26	HAES819	Hawaii	11.93	18.94	34.95	2.48	75.28	0.0289	0.0358	0.5541	46
66	Guire 1	China	12	17.04	34.05	2.51	76.81	0.0291	0.0359	0.5526	47
42	HAES904	Hawaii	12.43	15.35	32.2	1.82	74.43	0.0297	0.0365	0.5514	48
206	YXC3	China	11.7	20.82	30.93	2.39	75.85	0.0296	0.0349	0.5412	49
189	Ke2	China	11.35	29.21	26.79	2.48	74.34	0.0302	0.0356	0.5406	50
178	Lei5	China	11.6	22.18	28.82	2.25	73.36	0.0301	0.0345	0.5342	51
81	AY13	Australia	11.88	14.11	36.57	2.13	72.88	0.0309	0.035	0.5311	52
164	JH76	China	11.39	24.19	26.91	2.4	75.97	0.0305	0.0343	0.5294	53
120	JH27	China	11.29	25.55	26.13	2.17	72.59	0.0312	0.0339	0.5214	54
3	HAES246	Hawaii	11.59	17.7	31.97	1.82	74.3	0.0318	0.0336	0.5139	55
215	MT10	China	11.13	20.85	32.28	2.34	73.82	0.0316	0.0329	0.5102	56
8	HAES660	Hawaii	11.92	13.2	31.16	1.29	73.05	0.0334	0.0343	0.5067	57
98	Nanya 2	China	10.83	23.79	27.68	2.59	74.26	0.0321	0.0327	0.5041	58
173	JHG6	China	11.1	19.22	30.66	2.23	76.12	0.0323	0.0323	0.5004	59
108	JH13	China	10.84	22	31.39	2.42	76.4	0.0323	0.0322	0.499	60
86	AY17	Australia	11.36	15.34	33.48	1.7	72.05	0.0333	0.0326	0.4944	61
186	MD3	China	11.02	18.41	31.4	2	72.11	0.0331	0.0318	0.4897	62
103	JH07	China	10.54	19.84	36.92	2.72	73.44	0.0331	0.0317	0.4896	63
162	JH74	China	10.3	23.1	31.88	2.83	73.21	0.0336	0.0314	0.4833	64
63	Nanya 1	China	9.99	25.31	32.33	2.8	74.04	0.0345	0.0309	0.4723	65
112	JH18	China	10.19	24.02	32.29	2.46	75.17	0.0343	0.0305	0.4713	66
83	AY15	Australia	10.23	17.99	36.49	2.82	75.86	0.0345	0.0306	0.4705	67
177	Lei4	China	10.18	20.44	36.61	2.61	75.47	0.0344	0.0303	0.4685	68
2	Guang 11	China	10.63	17.85	30.5	1.96	73.8	0.0347	0.0302	0.4653	69
212	WD37	China	10.64	17.82	27.96	2	75.16	0.0347	0.0301	0.4646	70
116	JH23	China	10.02	26.54	24.71	2.53	72.48	0.0351	0.0304	0.4644	71
117	JH24	China	10.33	20.18	31.12	2.29	71.75	0.0346	0.0299	0.4635	72
15	HAES780	Hawaii	10.32	15.92	39.27	2.4	74.25	0.035	0.0301	0.4625	73
16	HAES783	Hawaii	10.24	17.92	36.93	2.36	75.63	0.035	0.0298	0.4598	74
200	Yukun1	China	10.25	20.33	29.4	2.35	73.91	0.0349	0.0297	0.4597	75
100	VN01	Australia	9.72	23.91	34.97	2.78	75.11	0.0355	0.0298	0.4562	76
121	JH28	China	10.33	17.14	35.05	2.14	74.81	0.0353	0.0296	0.456	77
45	HAES918	Hawaii	9.58	19.05	43.96	2.97	74.05	0.0363	0.0297	0.4499	78
85	AY16	Australia	9.3	18.44	46.41	3.41	73.28	0.0372	0.0303	0.4486	79
179	Lei6	China	9.56	25.45	30.53	2.66	73.24	0.0363	0.0291	0.4453	80
77	AY10	Australia	9.98	19.01	30.84	2.15	75.23	0.0363	0.0283	0.4379	81
202	DS1	China	9.13	23.84	35.69	3.14	75.53	0.0374	0.0289	0.4359	82
159	JH71	China	9.48	25.94	27.26	2.43	75.13	0.037	0.0284	0.4339	83
75	AY08	Australia	9.32	18.64	42.24	2.95	72.76	0.0373	0.0286	0.4336	84
174	Lei1	China	9.87	18.23	28.71	2.07	74.21	0.0371	0.0275	0.4264	85
251	C 4	Australia	9.5	17.31	32.29	2.77	76.24	0.0374	0.0277	0.4256	86
44	HAES915	Hawaii	10.05	15.97	30.29	1.56	72.83	0.0379	0.0276	0.4215	87

41	HAES900	Hawaii	9.08	26.07	26.81	2.71	76.27	0.0381	0.0277	0.4209	88
70	AY04	Australia	9.61	16.05	36.57	2.28	76.98	0.0377	0.0273	0.4198	89
7	HAES508	Hawaii	9.41	18.3	37.22	2.46	77.12	0.0377	0.0272	0.4192	90
17	HAES788	Hawaii	9.12	21.9	35.34	2.63	76.33	0.038	0.0271	0.4161	91
158	JH70	China	9.89	13.03	35.71	1.62	75.52	0.0388	0.0273	0.4132	92
218	WD34	China	9.42	15.9	39.9	2.14	75.63	0.0385	0.0267	0.4095	93
90	AY20	Australia	9.06	20.47	37.67	2.57	73	0.0384	0.0267	0.4095	94
165	JH77	China	9.58	19.1	25.06	1.93	72.32	0.0384	0.0263	0.4067	95
32	HAES842	Hawaii	9.17	16.5	36.67	2.43	74.65	0.0389	0.0261	0.4014	96
22	HAES812	Hawaii	9.41	14.97	36.4	2.05	75.9	0.039	0.0261	0.4013	97
139	JH50	China	9.3	21.95	26.59	1.93	73.38	0.0388	0.0259	0.4007	98
87	AY18	Australia	8.91	19.82	37.98	2.55	74.85	0.0391	0.026	0.4	99
21	HAES800	Hawaii	9.01	22.14	33.13	2.3	74.48	0.0389	0.0259	0.3999	100
187	MD4	China	9.09	18.38	27.2	2.09	73.6	0.0397	0.0248	0.384	101
123	JH31	China	8.2	24.25	34.24	2.9	75.23	0.0409	0.0254	0.3831	102
82	AY14	Australia	9.07	14.52	33.19	2.04	74.5	0.0404	0.0246	0.379	103
157	JH69	China	8.9	18.79	30.14	2.14	74.53	0.0401	0.0244	0.3783	104
88	AY19	Australia	8.52	17.92	38.19	2.45	76.48	0.0409	0.0243	0.3725	105
12	HAES762	Hawaii	8.82	19.65	29.33	1.93	74.68	0.0406	0.0239	0.3708	106
190	Ke3	China	8.66	19.87	34.43	2.05	74.45	0.0407	0.024	0.3706	107
161	JH73	China	9.08	17.06	26.2	1.68	75.44	0.0408	0.024	0.3706	108
36	HAES854	Hawaii	8.81	12.7	41.04	1.97	74.72	0.0416	0.0244	0.3692	109
60	H3	Hawaii	8.99	19.9	20.75	1.71	71.74	0.041	0.024	0.3692	110
143	JH54	China	8.37	18.36	36.61	2.36	72.91	0.0415	0.0235	0.3616	111
169	JHG1	China	8.3	17.93	32.46	2.6	73.72	0.0417	0.0234	0.359	112
104	JH08	China	8.59	20.44	28.63	1.81	72.82	0.0416	0.0231	0.3573	113
133	JH42	China	8.7	13.43	38.36	1.7	76	0.0424	0.0234	0.3558	114
194	Bai2	China	8.27	21.93	28.24	2.12	75.64	0.042	0.0228	0.3514	115
29	HAES828	Hawaii	8.27	14.9	39.56	2.25	76.72	0.0426	0.0229	0.3496	116
79	IY01	Australia	8.39	15.42	36.4	1.87	73.99	0.0427	0.0224	0.3437	117
101	Cold resistant	China	7.7	22.48	32.16	2.47	76.42	0.0434	0.0221	0.3374	118
23	HAES814	Hawaii	8.2	15.15	34.95	2.07	76.6	0.0432	0.0218	0.336	119
50	D	Australia	7.81	22.25	30.37	2.23	72.94	0.0434	0.0217	0.3336	120
65	800 TCS	China	7.53	21.88	34.41	2.55	74.89	0.0439	0.0218	0.3321	121
102	JH05	China	6.83	25.04	33.29	3.06	74.49	0.0459	0.0224	0.3279	122
209	MT20	China	7.87	20.58	33.19	1.91	75.3	0.0437	0.0212	0.3264	123
80	AY12	Australia	7.51	15.75	40.94	2.6	76.76	0.0447	0.0215	0.3249	124
76	AY09	Australia	6.98	19.6	38.96	2.85	77.57	0.0458	0.0211	0.315	125
91	AY21	Australia	6.64	21.78	37.17	2.87	73.2	0.0468	0.0206	0.3055	126
138	JH48	China	6.87	22.15	28.01	2.33	75.51	0.0467	0.0188	0.2868	127
156	JH67	China	6.74	22.17	31.14	2.16	74.28	0.0473	0.0183	0.2791	128
125	JH34	China	7.05	21.1	26.91	1.89	74.77	0.0468	0.0181	0.2784	129
62	HW1	Hawaii	7.05	13.85	35.39	2.03	76.05	0.0475	0.0179	0.2735	130
208	MT5	China	5.78	21.82	35.15	2.87	76.39	0.05	0.0182	0.2672	131
184	MD1	China	4.65	22.23	34.31	2.96	73.76	0.0541	0.0164	0.2332	132

205	YXC1	China	6.13	18.89	29.64	1.93	71.82	0.0502	0.0148	0.2279	133
242	38 seedling 2	China	5.74	16.19	37.82	2.27	76.51	0.0513	0.015	0.2267	134
74	AY07	Australia	4.85	17.54	37.82	2.66	72.26	0.054	0.0146	0.2129	135
126	JH35	China	5.59	20.62	27.01	1.92	74.74	0.052	0.0134	0.2053	136
146	JH57	China	4.86	24.15	24.83	2.11	73.24	0.0542	0.0136	0.2	137
223	HY03	Hawaii	3.94	18.15	33.49	2.44	75.66	0.0575	0.0115	0.1667	138
67	HY01	Hawaii	2.78	15.71	35.73	2.21	76.32	0.0621	0.0094	0.1314	139

Note: C16:1 (%), palmitoleic acid content; FW (g), fruit fresh weight; KR (%), kernel recovery; KW (g), single dry kernel weight; FAT (%), crude fat content; D+ and D-, Euclidean distances from the positive and negative ideal solutions, respectively; C, relative closeness coefficient. All 139 accessions were ranked; the top 10 are presented in Table 5 of the main text.
